# Dendritic domain-specific sampling of long-range axons shapes feedforward and feedback connectivity of L5 neurons

**DOI:** 10.1523/JNEUROSCI.1620-21.2022

**Published:** 2022-03-03

**Authors:** Alessandro R. Galloni, Zhiwen Ye, Ede Rancz

**Affiliations:** 1 The Francis Crick Institute, London NW1 1AT, United Kingdom; 2 University College London, WC1E 6BT, United Kingdom

## Abstract

Feedforward and feedback pathways interact in specific dendritic domains to enable cognitive functions such as predictive processing and learning. Based on axonal projections, hierarchically lower areas are thought to form synapses primarily on dendrites in middle cortical layers, while higher-order areas are posited to target dendrites in layer 1 and in deep layers. However, the extent to which functional synapses form in regions of axo-dendritic overlap has not been extensively studied. Here, we use viral tracing in the secondary visual cortex of male mice to map brain-wide inputs to thick-tufted layer 5 pyramidal neurons. Furthermore, we provide a comprehensive map of input locations through subcellular optogenetic circuit mapping. We show that input pathways target distinct dendritic domains with far greater specificity than appears from their axonal branching, often deviating substantially from the canonical patterns. Common assumptions regarding the dendrite-level interaction of feedforward and feedback inputs may thus need revisiting.

## Introduction

Hierarchical connectivity between cortical areas is often considered a central organizing principle underlying computations in the brain (Harris et al 2019, Vezoli et al 2021). An important aspect of this hierarchy is the interaction between feedforward (FF) and feedback (FB) signals, allowing neurons closer to the sensory input to adapt their responses based on high-level knowledge and objectives. How FF and FB signals are combined within individual neurons is an important unresolved problem with implications for our understanding of computation in both biological and artificial networks (Aru et al 2020, Guerguiev et al 2017, Larkum 2013).

In areas close to the sensory periphery, like the primary visual cortex (VISp), inputs can easily be designated as FF and FB. However, the recurrent nature of intra- and thalamocortical connectivity makes it more difficult to unambiguously categorize inputs in secondary sensory and associative areas. How then are FF and FB projections defined? Axonal pathways to visual cortical areas have distinctive laminar projection patterns that are broadly aligned with the hierarchy inferred from visual responses of neurons in each region. Specifically, FF projections mostly terminate in middle layers (particularly L4), while FB projections primarily target L1 and to a lesser extent deeper layers (Rockland & Pandya 1979). Similar projection patterns also appear in many other higher-level areas and have thus been used as a proxy to describe their hierarchical relationships (D’Souza et al 2020, Felleman & Van Essen 1991, Harris et al 2019, Wang et al 2020b).

At the single-cell level, these projection patterns imply that FF and FB projections may converge on different dendritic domains in individual neurons whose dendrites span multiple layers. Thick-tufted layer 5 (ttL5) neurons in particular have large dendritic trees spanning all cortical layers and biophysical properties that support highly non-linear integration of inputs, and are thus at the center of many theories of hierarchical computation in the brain (Guerguiev et al 2017, Larkum 2013, Payeur et al 2021). A common feature in these theories is FF connections targeting basal dendrites and FB connections synapsing onto the apical tuft in L1. This is based on the assumption that synapses form in the regions of greatest overlap between axons and dendrites — a principle known as Peters’ rule (Rees et al 2017). Projections, however, do not guarantee functional connections. While at the level of local interneuron connectivity there is some support for Peters’ rule (Fino & Yuste 2011, Packer et al 2013, Rieubland et al 2014), its general applicability has been refuted by dense anatomical reconstructions of retinal (Briggman et al 2011, Helmstaedter et al 2013, Kim et al 2014, Krishnaswamy et al 2015) and local excitatory cortical circuits (Kasthuri et al 2015, Lee et al 2016). Poor prediction of dendritic input by axonal distribution has been shown for some long-range connections (Little & Carter 2012, Petreanu et al 2009), but little is known about the general adherence to Peters’ rule at the level of long-range connectivity as it hasn’t been systematically studied.

To investigate how Peters’ rule applies to long-range projections, subcellular channelrhodopsin-assisted circuit mapping (sCRACM; Petreanu et al 2009) can be used. By selectively stimulating axons from specific input areas at different locations, highly specific dendritic targeting can be revealed (Anastasiades et al 2021, Yamawaki et al 2019). Here we used a combination of monosynaptically restricted rabies tracing (Kim et al 2015, Reardon et al 2016) and sCRACM recordings to comprehensively describe the functional input connectivity to thick-tufted layer 5 (ttL5) pyramidal neurons in medial secondary visual cortex and test whether Peters’ rule is valid for this neural population. We find that canonical FF and FB projection profiles do not match functional subcellular input maps, and thus hierarchical connectivity motifs should not be assumed based on axon and dendrite distributions alone.

## Materials and Methods

### Animals

All animal experiments were prospectively approved by the local ethics panel of the Francis Crick Institute (previously National Institute for Medical Research) and the UK Home Office under the Animals (Scientific Procedures) Act 1986 (PPL: 70/8935). Tg(Colgalt2-Cre)NF107Gsat (RRID:MMRRC_036504-UCD) mice crossed with the Ai14 reported line expressing tdTomato (RRID:IMSR_JAX:007908) were used throughout this work. Tg(Rbp4-Cre)KL100Gsat/Mmucd (RRID:MMRRC_031125-UCD) mice were used to establish the efficacy of the cre-off approach. As only male mice are transgenic in the Colgalt2-Cre line, all experiments were done on male animals.

### Viruses

EnvA-CVS-N2c^∆G^-mCherry rabies virus, and adeno associated viruses expressing TVA and EGFP (AAV8-EF1a-flex-GT), N2c glycoprotein (AAV1-Syn-flex-H2B-N2CG), or Cre-OFF Chronos-GFP (AAV1-EF1-CreOff-Chronos-GFP) were a generous gift of Molly Strom and Troy Margrie. Chronos-GFP (also called ShChR) expressing adeno associated virus (rAAV1-Syn-Chronos-GFP) was obtained from the UNC Vector Core.

### Surgical procedures

Surgeries were performed on mice aged 3-8 weeks using aseptic technique under isoflurane (2-4%) anesthesia and analgesia (meloxicam 2 mg/kg and buprenorphine 0.1 mg/kg). The animals were head-fixed in a stereotaxic frame and a small hole (0.5-0.7 mm) was drilled in the skull above the injection site. Viruses were injected using a Nanoject III delivery system (Drummond Scientific) at 0.4 nl/s.

For rabies virus tracing experiments, a 1:2 mixture of TVA and N2c glycoprotein expressing cre-dependent AAVs (10-20 nL) was injected (AP: lambda point - 0.8 mm, ML: 1.6 mm, DV: 0.6 mm). Rabies virus (50-100 nl) was injected 5-7 days later. Ten to twelve days later, animals were transcardially perfused under terminal anesthesia with cold phosphate-buffer (PB, 0.1 M) followed by 4% paraformaldehyde (PFA) in PB (0.1 M).

For the sCRACM experiments, Chronos-GFP expressing AAV was injected into one of the identified presynaptic regions. The range of stereotaxic coordinates - targeted to the location of the densest rabies labelling in each input region - are listed in Table 1. In V2M Cre-OFF Chronos-GFP was instead used to avoid expression in the recorded Colgalt2-Cre neurons. For injections into LP, the Chronos-GFP virus was diluted by 10-fold in sterile cortex buffer before injection to eliminate retrograde labelling of V2M neurons.

### Data acquisition and analysis for rabies tracing experiments

Brain samples were embedded in 4-5% agarose (Sigma-Aldrich: 9012-36-6) in 0.1M PB and imaged using serial two-photon tomography (Han et al 2018, Osten & Margrie 2013, Ragan et al 2012). Eight optical sections were imaged every 5 µm with 1.2 µm x 1.2 µm lateral resolution, after which a 40µm physical section was removed. Excitation was provided by a pulsed femto-second laser at 800 nm wavelength (MaiTai eHP, Spectraphysics). Images were acquired through a 16X, 0.8 NA objective (Nikon MRP07220) in three channels (green, red, blue) using photomultiplier tubes. Image tiles for each channel and optical plane were stitched together using custom-written MATLAB scripts (https://github.com/SainsburyWellcomeCentre/StitchIt). For cell detection, full resolution images were first filtered with a Gaussian blur (sigma = 1) using Fiji (ImageJ 1.52e) to reduce imaging noise. The open-source package “cellfinder” (Tyson et al 2020) was used for cell candidate detection and classification. Automated mouse atlas propagation (Niedworok et al 2016) was used for registration and segmentation (Allen CCFv3; Wang et al 2020a). For cell density visualization, cell coordinates were reversetransformed onto the Allen CCFv3 space using Elastix (Klein et al 2010).

### Acute slice preparation and electrophysiological recordings

Adult mice were anaesthetized with isoflurane and decapitated at least 17 days after the viral injection. The brain was rapidly removed and placed in oxygenated ice-cold slicing solution containing (in mM): 125 sucrose, 62.5 NaCl, 2.5 KCl, 1.25 NaH_2_PO_4_, 26 NaHCO_3_, 2 MgCl_2_, 1 CaCl_2_, 25 dextrose; osmolarity 340–350 mOsm. Coronal slices (300 µm) containing V2M were prepared using a vibrating blade microtome (Leica VT1200S). Slices were kept submerged in artificial cerebrospinal fluid (ACSF, containing in mM: 125 NaCl, 2.5 KCl, 1.25 NaH_2_PO_4_, 26 NaHCO_3_, 1 MgCl_2_, 2 CaCl_2_, 25 dextrose; osmolarity 308–312 mOsm) at 35°C for the first 30–60 min after slicing, then at room temperature (22°C). All solutions were continuously bubbled with carbogen (95% O2 / 5% CO2).

The recording chamber was perfused at a rate of 6 mL/min with ACSF at room temperature (22°C). To prevent axonal spike propagation and enhance responses to optical stimulation, 1 µM tetrodotoxin (TTX) and 100 µM 4-aminopyridin (4-AP) were added to the recording ACSF. Filamented borosilicate glass micropipettes were pulled and heat-polished using a horizontal puller (Zeitz DMZ Universal Electrode Puller) to obtain an electrode resistance of 3-6 MΩ. The glass electrodes were filled with the internal solution containing (in mM): 120 CsMeSO_3_ (CH_3_O_3_SCs), 3 CsCl, 10 HEPES, 1 EGTA, 4 Na_2_ATP, 0.3 NaGTP, 5 Na_2_-phosphocreatine (C_4_H_8_N_3_O_5_PNa_2_), 3.5 QX-314 chloride, 0.5 % (w/v) biocytin hydrochloride, 50 µM Alexa Fluor 488 hydrazide; osmolarity 290–295 mOsm; pH adjusted to 7.3 with CsOH.

Visually guided whole-cell patch-clamp recordings from tdTomato-labelled Colgalt2-Cre neurons in V2M were performed using a Scientifica SliceScope Pro 3000 microscope equipped with a 40x/0.8 NA objective and an infrared (IR) Dodt Gradient Contrast system. A CoolLED pE-4000 light source (550 nm) was used to visualize fluorescence in Cre-expressing neurons. Before each recording, the apical dendrite was visually inspected to verify that it was not cut and could be seen to descend at a shallow angle into the slice. For neurons that were successfully filled with biocytin during the recording, we also confirmed that all dendrites, except a small number of basal and tuft dendrites extending directly towards the slice surface, were intact. Recordings were made with a Multiclamp 700B amplifier (Molecular Devices) in voltage-clamp configuration with a holding potential of -70 mV. Filtered signals (8kHz low-pass) were digitized at 20 kHz with a National Instruments DAQ board (PCIe-6323). Acquisition and stimulus protocols were generated in Igor Pro (Wavemetrics) with the NeuroMatic software package (Rothman & Silver 2018). Throughout each recording, series resistance compensation was set to the highest value possible without inducing oscillations in the cell (typically between 40 and 75%). Recordings with series resistance larger than 40 MΩ were discarded.

### Patterned optogenetic stimulation

Optical stimulation was implemented using a digital micromirror device (DMD) coupled to a 463 nm CW laser (Polygon 400, Mightex Systems). The stimulus consisted of a 1000 x 500 µm grid divided into 24 x 12 spots of light (41.7 µm x 41.7 µm square) delivered through a 5x/0.15 NA objective (Olympus MPlanFL N). The grid was centered on the soma and aligned to the pia orthogonal to the apical dendrite. The laser output associated with each spot was measured (PM100D and S121C, Thorlabs) and adjusted to obtain a measured power of approximately 300 µW (173 mW/mm2). Optical stimuli were delivered for 1 ms at 10 Hz in a pseudo-random sequence designed to maximize the distance between consecutive spots and the time between stimulation of neighboring spots. Each recording trial consisted of a single repetition of all 288 stimuli followed by a full-field stimulus, in which all stimulation spots were illuminated simultaneously for 1 ms. 5-20 trials were recorded, with 30s pauses between trials, making the interval between consecutive stimulation of the same spot 60s. An image of the recorded cell (filled with Alexa Fluor 488) relative to the stimulation grid was used during analysis to align the recorded sCRACM heatmap with the location of the pia or soma.

### Immunohistochemistry & morphological reconstructions

After recording, slices were fixed overnight at 4°C in a 4% paraformaldehyde solution and were subsequently kept in PBS. Slices were stained with DAPI (5 µg/mL) for 10 min, mounted on glass slides and images were acquired with either a confocal microscope for high-resolution images (Leica SP5; objective: 20x/0.7NA or 10x/0.4NA; pinhole size: 1 airy unit) or a slide scanner for visualizing injection sites (Olympus VS120, objective: 4x/0.16NA). Image processing was done with the FIJI software package. For the detailed morphological analysis, a subset of neurons, selected based on the quality and completeness of staining, was reconstructed in full through the LMtrace service of https://ariadne.ai/lmtrace.

### Comparison of axonal projection patterns to VISam, VISpm and RSPagl

We have extracted layer-wise axonal projection data for the 6 input areas (including all genotypes, ACA n=33, ATN n=11, LP n=10, ORB n=11, RSPg n=17, VISp n=60) from the Allen Mouse Brain Connectivity database (© 2011 Allen Institute for Brain Science. Allen Mouse Brain Connectivity Atlas. Available from: https://connectivity.brain-map.org/). Projection energy followed the same layer-wise pattern across the three target areas for all input areas (p > 0.05, 2-way ANOVA with Tukey’s post hoc test) except for ACA (RSPagl vs VISpm p = 0.001; VISam vs VISpm p = 0.016). When only wild-type data was considered, no statistical difference between target areas was detected (p > 0.05, 2-way ANOVA with Tukey’s post hoc test; n = ACA 5, ATN 4, LP 2, ORB 2, RSPg 2, VISp 21).

### Data analysis

Analysis and data visualization were performed with custom scripts written in Igor Pro and MATLAB. Recordings were baselined in a 40 ms window before each stimulus and averaged across trials, and the peak and area (equivalent to the charge) of the evoked currents were measured in a 50 ms window after the stimulus. Currents with peaks greater than seven times the standard deviation of the baseline were included in the analysis. Occasional cells showing low latency, direct photocurrent presumably resulting from retrograde infection were excluded from the analysis (ATN - 0/16; ORB - 0/18; ACA - 1/28; VISp -0/14; V2M - 1/23; RSPg - 4/30; LP - 0/8 with 1:10 dilution and 11/17 with undiluted virus injection). Subsequently, recorded cells falling outside the borders of V2M were excluded. Heatmaps were normalized to the peak synaptic charge for each cell, aligned horizontally by soma location and vertically by either soma or pia location, and then averaged. Somas were localized to one quadrant of a single stimulation spot, resulting in a pixel dimension of 20.8 x 20.8 µm. Note, however, that the effective resolution is limited by light scattering and by the spread of voltage along stimulated axons. Previous studies have indicated that these factors limit the actual sCRACM resolution to approximately 60 µm (Petreanu et al 2009). Input maps for individual cells were convolved with the average ttL5 morphology obtained from 11 reconstructed V2M Colgalt2-Cre neurons. The apical tuft, oblique (including the apical trunk), and basal dendrites were manually labelled, and the total dendritic length was quantified in 10 µm bins along the apical dendrite axis using Neurolucida 360. The resulting dendrite profiles were aligned, averaged, and scaled to the soma-pia distance for each recorded cell. For testing Peters’ rule, i.e. to enable a direct comparison with the pia-aligned axon and sCRACM maps, morphologies were pia-aligned before averaging. Axon and dendrite distributions were normalized to their respective peaks and multiplied, resulting in large values for predicted input at locations containing both axons and dendrites.

### Experimental Design and Statistical Analyses

Sufficient sample sizes were estimated based on similar published experiments performed in other brain regions (Petreanu et al 2009, Young et al 2021). Data are presented are mean ± SEM unless specified otherwise. Statistical analyses were performed in Matlab and were corrected for multiple comparisons using the Benjamini-Hochberg procedure where appropriate. Statistical test used are specified and absolute p values are given.

## Results

To ensure recording from a homogeneous neuronal population, we used the Colgalt2-Cre mouse line which specifically labels subcortically projecting, thick-tufted layer 5 (ttL5) neurons (Groh et al 2010, Kim et al 2015). We focused our study on the medial secondary visual cortex (V2M) as higher order cortical regions are likely to receive a broader diversity of long-range inputs than primary sensory cortices. V2M is defined in the Mouse Brain In Stereotaxic Coordinates atlas (Franklin & Paxinos 2007, Kirkcaldie 2012) which can be used to guide viral injections. Furthermore, as this atlas is based on cytoarchitecture, V2M can be visually distinguished and selectively targeted in slice recordings, as has been done previously (Galloni et al 2020, Young et al 2021). For whole-brain rabies tracing, on the other hand, we adopted the more recently developed Allen Common Coordinate Framework (CCFv3, Wang et al 2020a). This atlas allowed us to localize individual neurons within 3D volumes of brain tissue, which is not possible using the Franklin & Paxinos atlas. Within the CCFv3, the V2M region corresponds to areas VISpm, VISam, and RSPagl (Lyamzin & Benucci 2019), all of which are known to be visually responsive (Garrett et al 2014, Powell et al 2020). Treating V2M as a uniform region for the purposes of this study was also supported by the Allen Mouse Brain Connectivity database, which shows that axonal projections to VISpm, VISam, and RSPagl are not substantially different (see Methods for details).

## Brain-wide input map to V2M ttL5 pyramidal neurons

**Figure 1:**
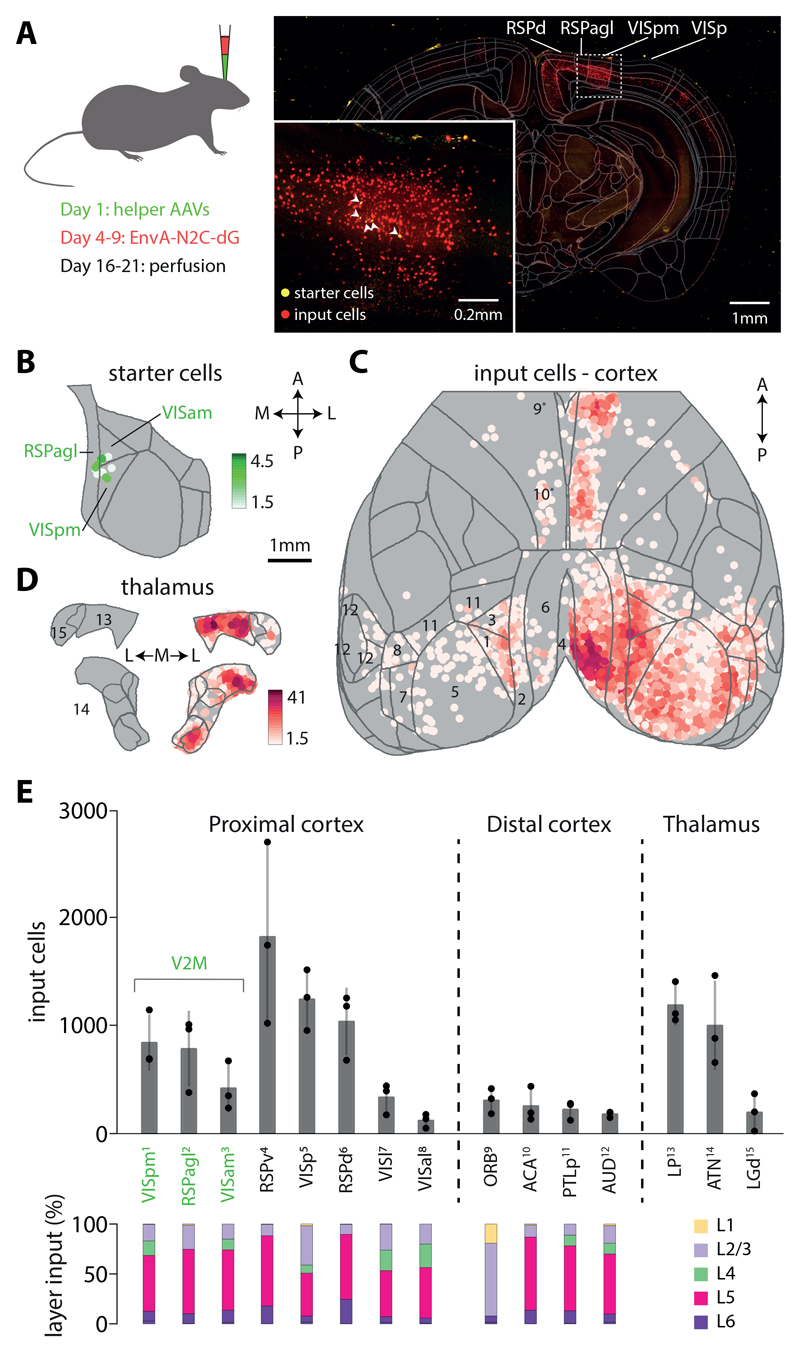
Whole-brain input map to V2M ttL5 neurons. **A.** Maximum intensity projection of a 200 µm thick coronal slab containing starter neurons, with Allen CCFv3 outlines overlayed. Inset shows starter area with higher magnification. **B.** Starter cell density map from an example experiment (same as in A). **C.** Cortical input cell density map projected onto the horizontal plane, same experiment as in A. **D.** Thalamic input cell density map projected onto two coronal planes, same experiment as in A. Density scales are in cells / 0.01 mm^2^; arrows represent anterior, posterior, medial and lateral directions. **E.** Input cell numbers for the most prominent input areas. Averages, standard deviation, and individual experiments are shown. Bottom: distribution of input cells across cortical layers. Named areas are marked with numbers in C and D, asterisks denote areas below the cortical surface. Area definitions and nomenclature according to the Allen CCFv3. Comprehensive cell counts for individual experiments can be found in Figure 1-1.

We employed a monosynaptically restricted rabies virus approach (Reardon et al 2016, Wickersham et al 2007) to generate a presynaptic input map of V2M ttL5 neurons. Briefly, a mix of adeno-associated viruses carrying floxed N2c G-protein, or TVA-receptor and EGFP genes were injected into V2M of Colgalt2-Cre mice under stereotaxic guidance. Four to nine days later, mCherry-expressing EnvA-CVS-N2c-∆G rabies virus was injected at the same location. After a further 12-13 days, brains were fixed and imaged using serial section 2-photon tomography (Figure 1A). The resultant datasets were registered to the Allen CCFv3 atlas and presynaptic cell bodies were detected and counted using an automated pipeline (see Methods for details).

Example images and cell density maps are shown in Figure 1. Starter cells were scattered across V2M (Figure 1B) while presynaptic input neurons were detected in a broad range of cortical and subcortical areas (Figure 1C,D). We have grouped the most prominent input areas into proximal cortex, distal cortex, and thalamus (Figure 1E). Most input cells were found locally in V2M and in the proximal cortical areas VISp and the granular retrosplenial cortex (RSPg, consisting of RSPd and RSPv). Orbitofrontal cortex (ORB) and the anterior cingulate area (ACA) provided the most numerous distal cortical inputs. Interestingly, while most cortical input cells were detected in the granular and infragranular layers, especially layer 5, input from ORB was almost exclusively from layer 2/3 (Figure 1E). Prominent thalamic inputs were also observed, originating mainly in the lateral posterior nucleus (LP) and anterior thalamic nuclei (ATN). Comprehensive cell counts for individual experiments can be found in Figure 1-1.

To understand the subcellular organization of the diverse input pathways, we chose to examine 7 prominent input areas. We grouped them in three categories based on their connectivity distance from the visual sensory periphery: VISp and V2M as FF input; RSPg, ACA and ORB as cortical FB input; and LP and ATN for thalamic FB connections. We designate local (V2M) input as FF, as ttL5 neurons are considered the outputs of the cortical column and show very limited local projections.

## Subcellular optogenetic input mapping reveals diverse targeting of dendritic domains by input areas

**Figure 2:**
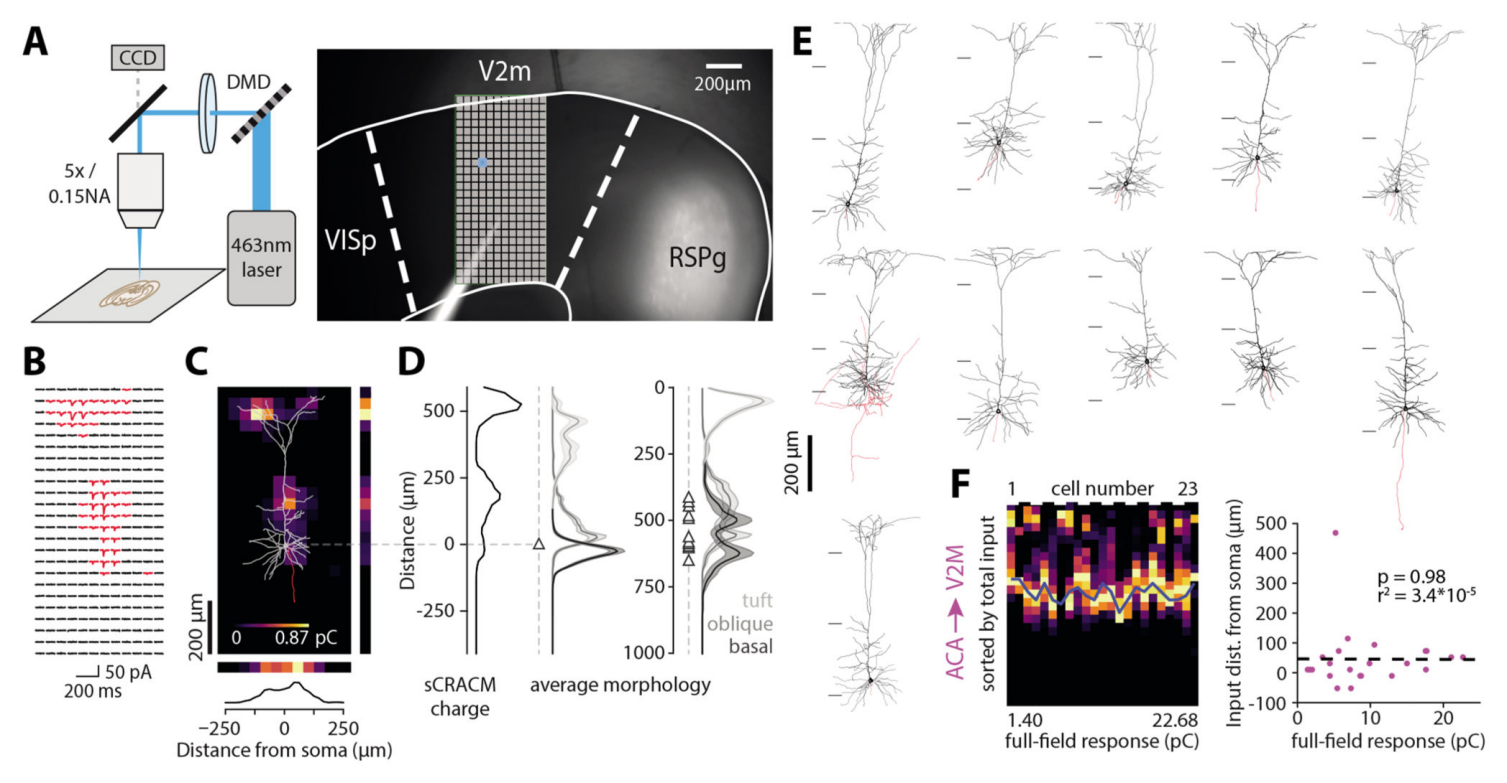
Using sCRACM to map subcellular connectivity. **A.** Experimental setup and micrograph showing a brain slice with Chronos expression in RSPg and recording pipette in V2M. The stimulation grid is overlaid, and an example spot is highlighted in blue. **B**. sCRACM recording of excitatory synaptic currents (red > 7 x baseline S.D.) from an example neuron. **C.** Charge heatmap corresponding to recording in B with the morphology of the recorded neuron overlaid. **D.** Left: normalized vertical profile of the input map in C. Middle: average soma-aligned morphology profile used for dendritic domain deconvolution. Right: averaged pia aligned morphological profile. Triangles represent soma location. **E.** Morphological reconstruction of 11 Colgalt2-cre neurons used for the average morphological profile. Black: dendrites; red: axons. Horizontal lines demarcate the border between L1-L2/3 and the top and bottom border of L5. **F.** Horizontal projections of ACA input to individual cells sorted by full-field stimulation response. Right: Location of the largest input peak versus full-field response. Dashed line is a linear fit.

To determine the spatial distribution of synaptic inputs to ttL5 neurons in V2M, we performed sCRACM experiments from selected input areas identified by the rabies tracing. Following expression of the optogenetic activator Chronos in different input areas (see methods for injection details), we made voltage-clamp recordings (at -70 mV) from tdTomato labelled (Colgalt2-Cre) ttL5 neurons in V2M using acute brain slices. Optical stimulation with a 463 nm laser was spatially targeted using a digital micromirror device (Figure 2A). Sodium and potassium channels were blocked using TTX (1µm) and 4-AP (100 µm) to ensure that evoked currents were restricted to directly stimulated nerve terminals and to enhance presynaptic release, respectively. The stimulus consisted of 24 x 12 spots of light in a 1000 x 500 µm grid aligned to the axis of the apical dendrite of the recorded neuron and covering the whole depth of cortex. We also quantified the total input from a given connection by recording synaptic currents evoked by full-field stimulation. To facilitate comparison between projections, we used the same laser intensity across all experiments.

Synaptic strength at each location was estimated by measuring the area of evoked synaptic currents (corresponding to charge; Figure 2B) and creating normalized 2D maps of the spatial distribution of inputs (Figure 2C). Individual maps were then aligned (either to the pia or soma) and averaged. To quantify the spatial location of inputs, we projected the average 2D maps in directions perpendicular (Figure 2C) or parallel with the apical dendrite (Figure 2D). Furthermore, we defined the spatial distribution of the three main dendritic compartments based on 11 morphologically reconstructed Colgalt2-Cre neurons (Figure 2E). Basal dendrites were defined as those originating at the soma, oblique dendrites as those originating from the apical trunk before the main bifurcation (including the apical trunk itself), and apical tuft dendrites as those originating after the bifurcation. As all three dendritic compartments have similar spine densities (Romand et al 2011), the horizontal projection of the average morphology was used to separate the contribution of each dendritic domain to the total synaptic input (Figure 2D).

One potential concern when recording distal synaptic currents from a somatic electrode is the effect of attenuation on detectability of currents. In neurons with weaker overall input, this might result in distal currents becoming too small to detect, thus biasing the input map towards the soma. We tested this by examining the correlation between the location of synaptic input and the total synaptic charge evoked by full-field stimulation (see Figure 2F for an example input area). No correlation was found for any of the recorded areas (p > 0.4), suggesting that the passive distance-dependent attenuation introduced no detection bias for distal inputs.

### Primary visual cortex

**Figure 3:**
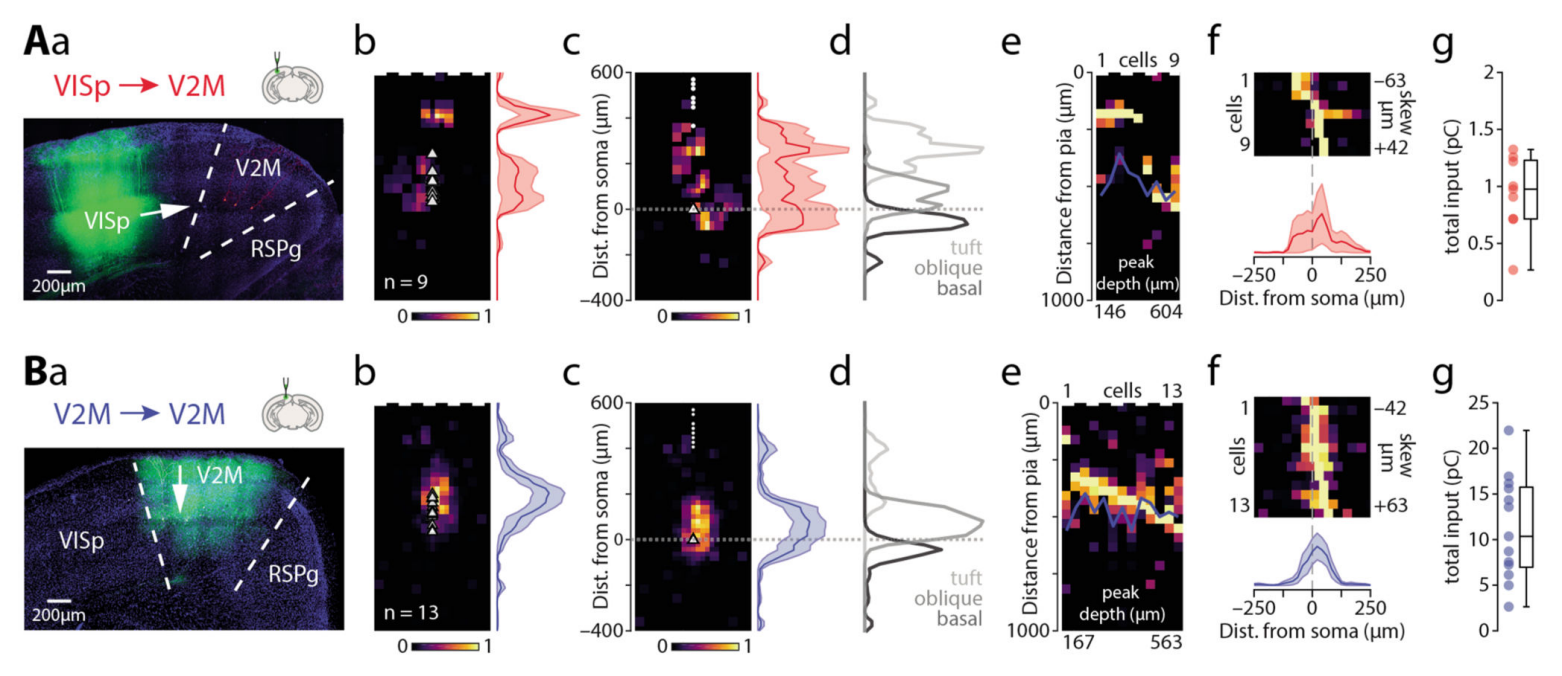
Subcellular connectivity maps of FF input areas. **A.** a: confocal image of a representative brain slice (blue = DAPI) showing the injection site in VISp (green) and recorded neurons in V2M (red). b: pia-aligned average sCRACM heatmap for VISp inputs. Triangles represent soma locations. The vertical profile indicates the normalized average and SEM of the input distributions across all recorded neurons. c: Same as in b but aligned on the soma location. Dots indicate pia locations. d: Normalized input magnitude deconvolved with the average morphology. Dotted line indicates soma location. e: Vertical projections of individual input maps sorted by the location of the peak input. f: Horizontal projections of individual input maps and their average. g: Box plot showing total input charge recorded during full-field stimulation. **B.** Same as in A but for Cre-off Chronos injections into V2M.

We first recorded optically evoked synaptic responses arising from VISp axons (n = 9 cells from 6 animals, average soma depth 507 ± 22 µm; Figure 3A). The apical tuft received 42% of the input, with a peak input located 188 µm from the pia (Figure 3Ab-d). The remaining input was spread between the oblique compartment, receiving 33%, and basal dendrites, receiving 26% of the total input (Table 2). More of the recorded neurons had the peak input in the apical compartment (n = 5 / 9) while 4 cells lacked apical input (Figure 3Ae). The horizontal input distribution showed a slight medial skew (towards RSPg), most prominent in the oblique (63 µm) and basal compartments (42 µm; Table 2, Figure 3Af). The total synaptic charge measured via the somatic recording following full-field stimulation was 0.93 ± 0.11 pC (Figure 3Ag). VISp thus provides moderate direct input to ttL5 neurons in V2M, primarily targeting the proximal part of the apical tuft (0.39 pC) with smaller input arriving to the oblique (0.30 pC) and basal (0.24 pC) compartments.

### Local input from V2M

To estimate the distribution of local input we used a Cre-off viral strategy, limiting Chronos expression to non-Colgalt2-Cre neurons (n = 13 cells from 4 animals, average soma depth 498 ± 15 µm; Figure 3B). When testing this strategy using the Rbp4-Cre line, which densely labels L5 pyramidal neurons, we found that only a very small proportion (3%) of Cre-positive cells expressed Chronos. The peak input was located close to the soma, at 396 µm from the pia (Figure 3Bb-d). The oblique compartment received the majority (62%) of this input, with the basal dendrites and apical tuft receiving 24% and 14%, respectively, of the total input (Table 2). For the majority of recorded neurons, the peak input occurred perisomatically (n = 12 / 13; Figure 3Be). The horizontal input distribution showed slight medial bias (-21 µm for all peaks; Table 2, Figure 3Bf). The total synaptic charge triggered by fullfield stimulation was 11.24 ± 1.56 pC (Figure 3Bg). Local neurons thus provide large direct input to ttL5 neurons in V2M, primarily targeting the oblique (6.96 pC) compartment, with smaller input arriving to the basal (2.68 pC) and tuft (1.6 pC) compartments.

### Granular retrosplenial area

**Figure 4:**
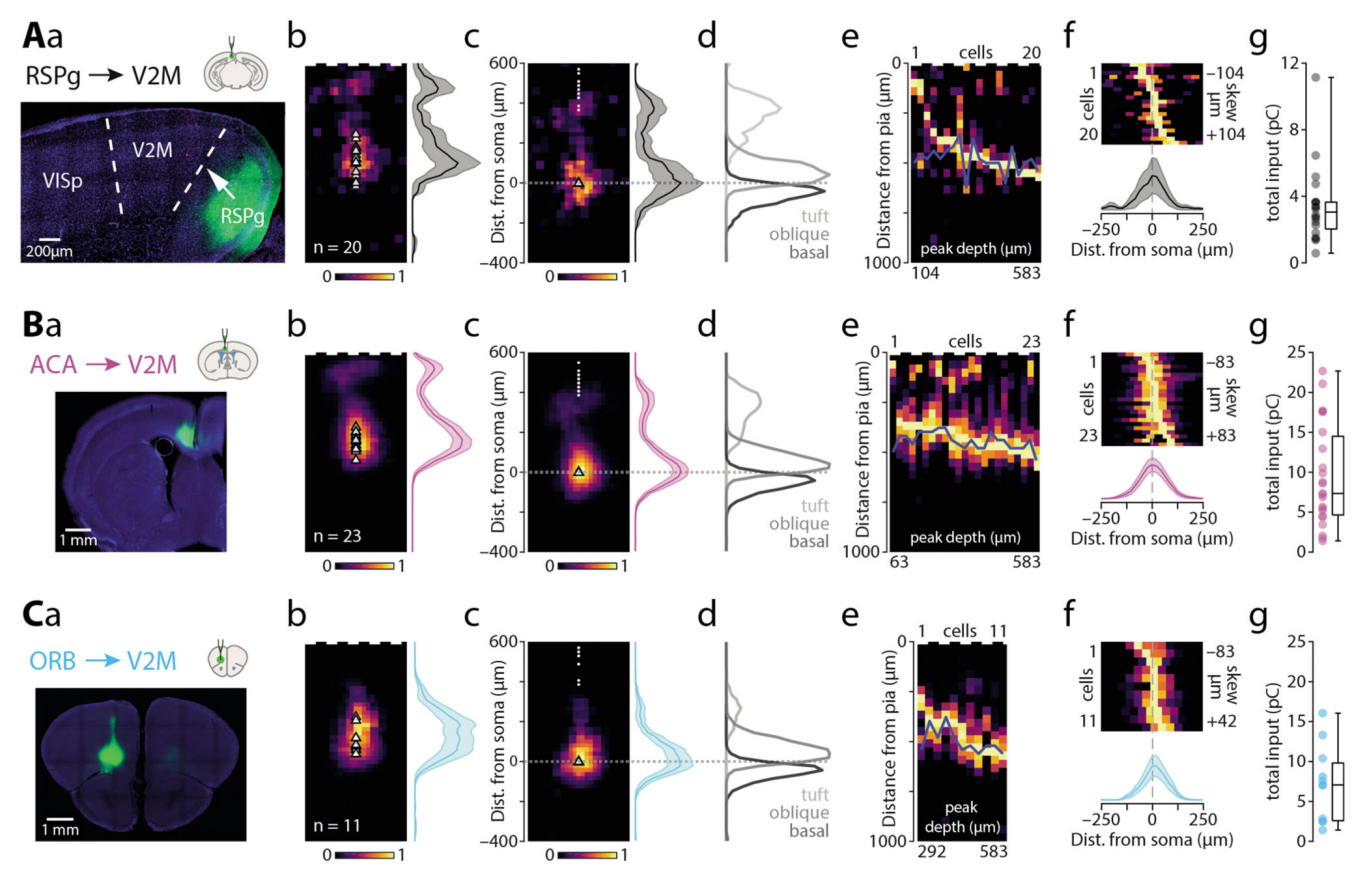
Subcellular connectivity maps of cortical FB areas. **A.** a: confocal image of a representative brain slice (blue = DAPI) showing the injection site in RSPg (green). b: pia-aligned average sCRACM heatmap for RSPg inputs. Triangles represent soma locations. The vertical profile indicates the normalized average and SEM of the input distributions across all recorded neurons. c: Same as in b but aligned on the soma location. Dots indicate pia locations. d: Normalized input magnitude deconvolved with the average morphology. Dotted line indicates soma location. e: Vertical projections of individual input maps sorted by the location of the peak input. f: Horizontal projections of individual input maps and their average. g: Box plot showing total input charge recorded during full-field stimulation. **B.** Same as in A but for Chronos injections into ACA. **C.** Same as in A but for Chronos injections into ORB.

Next, we recorded optically evoked synaptic responses arising from RSPg axons (n = 20 cells from 9 animals, average soma depth 503 ± 15 µm; Figure 4A). The overall input displayed a bimodal distribution peaking at 125 and 500 µm from the pia (Figure 4Ab-d). The apical tuft received 30% of the input, with the oblique compartment receiving 40% and basal dendrites 30% of the total input (Table 2). For the majority of recorded neurons, the peak input targeted the perisomatic dendrites (n = 18 / 20; Figure 4Ae). The horizontal input distribution showed slight medial bias (Table 2, Figure 4Af). Total synaptic charge triggered by full-field stimulation was 3.40 ± 0.51 pC (Figure 4Ag). RSPg thus provides a relatively moderate direct input to ttL5 neurons in V2M, targeting the oblique (1.36 pC), basal (1.04 pC) and apical tuft (1.01 pC) compartments to similar extent.

### Anterior cingulate area

Next, we recorded optically evoked synaptic responses arising from ACA axons (n = 23 cells from 5 animals, average soma depth 464 ± 9 µm; Figure 4B). The overall input was bimodal, peaking at 83 µm and 438 µm from the pia (Figure 4Bb-d). The apical tuft received 25% of the input, with the oblique compartment receiving 45% and basal dendrites 30% of the total input (Table 2). The majority of recorded neurons had the peak input located perisomatically (n = 22 / 23; Figure 4Be). The horizontal input distribution showed no medio-lateral bias (Table 2, Figure 4Bf). The total synaptic charge triggered by fullfield stimulation was 9.46 ± 1.32 pC (Figure 4Bg). ACA thus provides a large direct input to ttL5 neurons in V2M, primarily targeting the oblique (4.22 pC) compartment with smaller input arriving to the basal (2.83 pC) and most distal part of the apical tuft (2.41 pC).

### Orbitofrontal cortex

Optically evoked synaptic responses arising from ORB axons (n = 11 cells from 3 animals, average soma depth 521 ± 19 µm; Figure 4C) showed a strong perisomatic bias, with a peak at 417 µm from the pia (Figure 4Cb-d). The apical tuft received only 9% of all input, with the oblique compartment receiving 57% and basal dendrites 35% of the total input (Table 2). This distribution was also highly homogeneous across neurons, with almost all recorded neurons having their peak input in the perisomatic region (n = 11/11; Figure 4Ce). The horizontal input distribution showed no lateral bias (Table 2, Figure 4Cf). The total synaptic charge triggered by full-field stimulation was 7.16 ± 1.43 pC (Figure 4Cg). ORB thus provides a large direct input to ttL5 neurons in V2M, primarily targeting the oblique (4.05 pC) and basal (2.47 pC) compartments, with slight input arriving to the proximal part of the apical tuft (0.63 pC).

### Anterior thalamic nuclei

**Figure 5:**
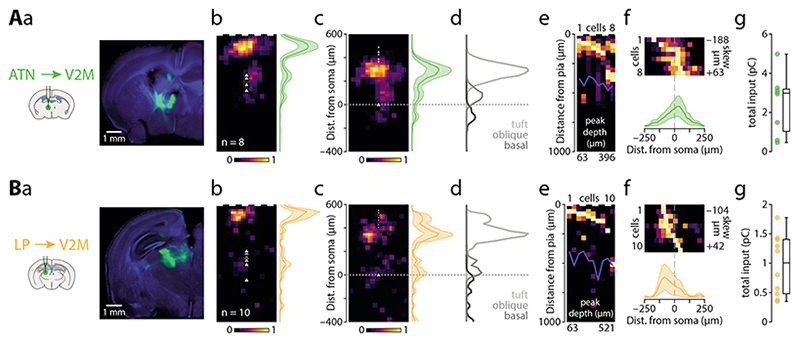
Subcellular connectivity maps of thalamic input areas. **A.** a: confocal image of a representative brain slice (blue = DAPI) showing the injection site in ATN (green). b: pia-aligned average sCRACM heatmap for ATN inputs. Triangles represent soma locations. The vertical profile indicates the normalized average and SEM of the input distributions across all recorded neurons. c: Same as in b but aligned on the soma location. Dots indicate pia locations. d: Normalized input magnitude deconvolved with the average morphology. Dotted line indicates soma location. e: Vertical projections of individual input maps sorted by the location of the peak input. f: Horizontal projections of individual input maps and their average. g: Box plot showing total input charge recorded during full-field stimulation. **B.** Same as in A but for Chronos injections in LP.

Next, we recorded optically evoked synaptic responses from thalamic axons, starting with the ATN (n = 8 cells from 3 animals, average soma depth 435 ± 15 µm; Figure 5A). This input had peaks at both 104 µm and 333 µm from the pia (Figure 5Ab-d). The apical tuft received the majority (75%) of the input, while the oblique compartment received 17% and basal dendrites a mere 8% of the total input (Table 2). The majority of recorded neurons had the peak input in the tuft compartment (n = 6 / 8) and while all cells had some tuft input, in 2/8 cells the input peak was located perisomatically (Figure 5Ae). The horizontal input distribution showed a medial bias (Table 2, Figure 5Af). The total synaptic charge triggered by full-field stimulation was 2.48 ± 0.54 pC (Figure 5Ag). ATN thus provides a moderate direct input to ttL5 neurons in V2M, primarily targeting the more distal part of the apical tuft (1.86 pC) while the oblique (0.42 pC) and the basal (0.21 pC) compartment received less input.

### Lateral posterior nucleus of the thalamus

Lastly, we recorded optically evoked synaptic responses arising from LP axons (n = 10 cells from 4 animals, average soma depth 500 ± 23 µm; Figure 5B). Due to excessive retrograde labelling resulting in direct photocurrent in the recorded V2M cells, a 1:10 dilution of virus was used for these injections and the absolute value of the evoked input is thus likely an underestimate. As with ATN axons, the LP input was strongly biased towards the most superficial part of the cortex and peaked at 63 µm from the pia (Figure 5Bb-d). The apical tuft received the vast majority (75%) of the input, with the oblique compartment receiving 15% and basal dendrites 10 % of the total input (Table 2). Most recorded neurons had the peak input in the tuft compartment (n = 9/10; Figure 5Be). The horizontal input distribution showed lateral bias (Table 2, Figure 5Bf). The total synaptic charge triggered by full-field stimulation was 0.97 ± 0.16 pC (Figure 5Bg). LP thus provides modest direct input to ttL5 neurons in V2M, primarily targeting the most distal part of the apical tuft (0.72 pC) compartment with smaller input arriving to the oblique (0.14 pC) and the basal (0.10 pC) compartments.

## Comparison of anatomical and functional connectivity maps

Having determined the spatial distribution of synapses for the main input areas, we next sought to directly compare this to what would be predicted from axo-dendritic overlap (i.e., from Peters’ rule). To determine axonal projection patterns from input areas to V2M, we have imaged the Chronos-eGFP labelled axons in a subset of the brain slices used for the sCRACM experiments using confocal microscopy.

**Figure 6:**
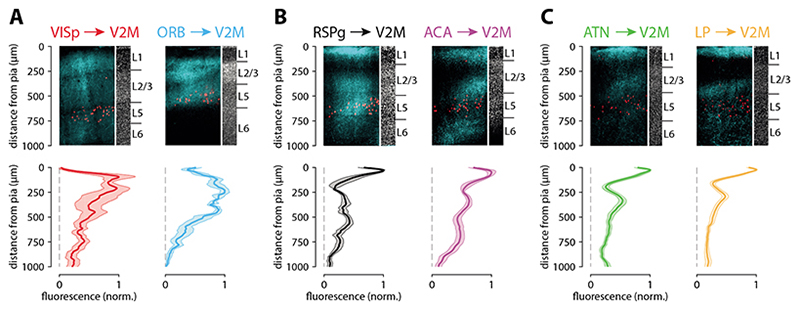
Axonal projection densities from different input areas. **A-C.** Top: example confocal images from V2M showing axonal projections from six input areas (cyan) and Colgalt2-Cre cell bodies (red). The corresponding DAPI staining shows variation in laminar depth. Bottom: average projection density profiles across the cortical depth averaged across 3-5 injections.

The spatial distribution of axons followed three basic patterns. Axons from VISp and ORB were densest in L2/3 and L5 while little projection was apparent in L1, reminiscent of the classical FF projection pattern (Figure 6A). In contrast, axons from RSPg and ACA showed an FB-like pattern with dense labelling in the middle part of L1 followed by sparse labelling in L2 and diffuse axons in layers 3, 5 and 6 (Figure 6B). The final group, which consists of the thalamic projections from LP and ATN, showed the classical FB pattern strongly innervating the external part of L1, with a secondary peak in L3, but little or no projections in layers 2, 5 and 6 (Figure 6C).

**Figure 7:**
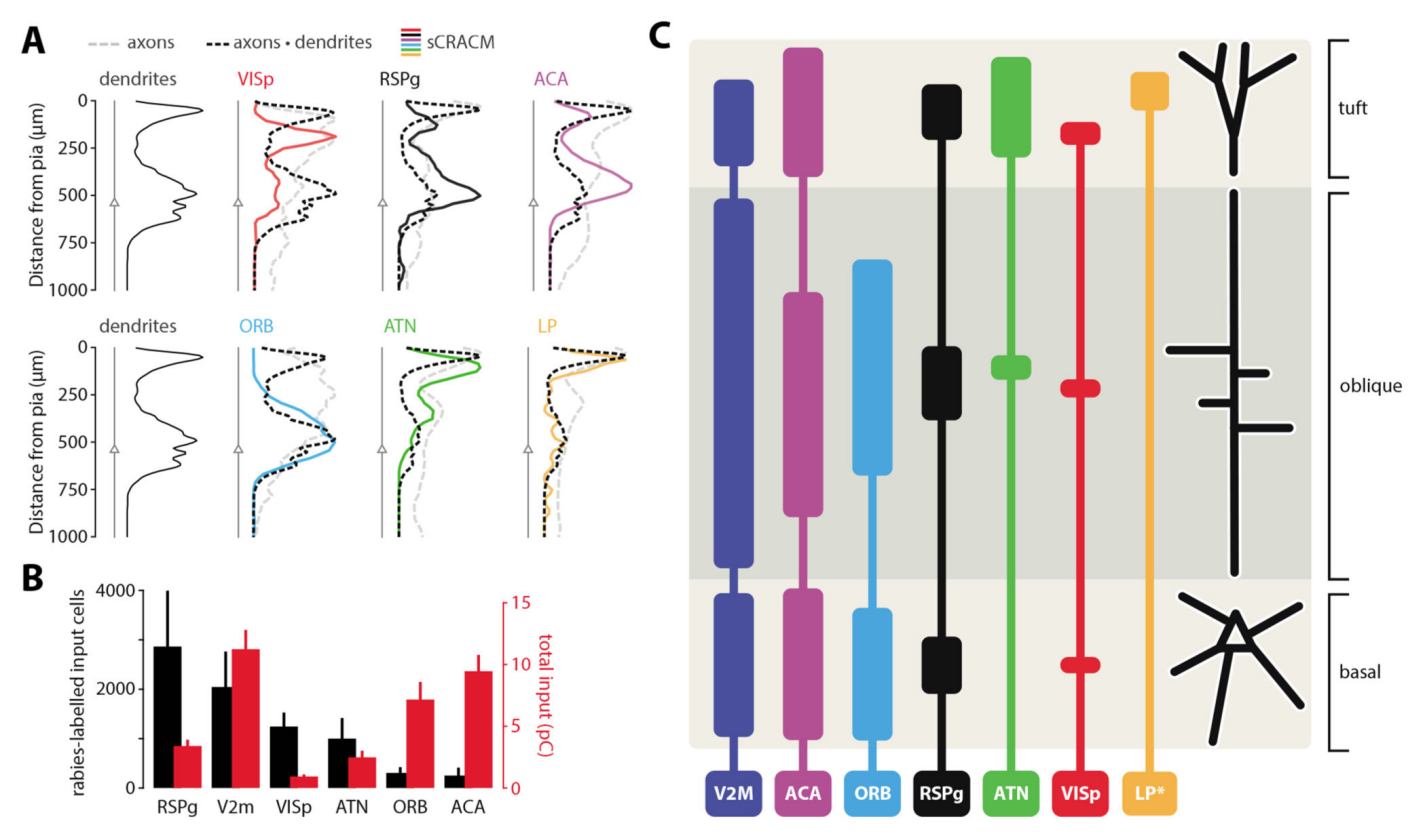
Comparison of different input maps. **A.** Axonal density distributions multiplied with dendritic morphology (dotted black lines) overlaid with pia-aligned synaptic input distributions (colored lines). Six input areas are shown. Triangles represent soma location in average morphology. **B.** Number of input cells across 6 areas established by rabies tracing (black) and total input charge recorded during full-field optogenetic stimulation (red). **C.** Schematic of excitatory synaptic input map to ttL5 pyramidal neurons. The height of bars represents input strength, while the center of each bar is aligned to the peak of the sCRACM map. The tuft input map was generated from pia aligned maps while oblique and basal maps from soma aligned maps. NB: LP input magnitude (*) is likely underestimated due to the lower virus titer used.

To accurately estimate morphological overlap between axons and dendrites, we multiplied the axonal projection maps with the average dendritic morphology, resulting in the predicted input distribution one would expect to see based on Peters’ rule. When overlaying this with the pia-aligned vertical sCRACM maps, the alignments between functional synapses and the axo-dendritic maps were diverse (Figure 7A). For some regions, like ORB perisomatic and LP tuft inputs, a clear correspondence could be seen between predicted and measured input distributions. A lesser degree of overlap can be seen in the VISp perisomatic or ACA tuft inputs. For other inputs, however, strong functional input could be detected where there is little overlap between dendrites and axons, such as at VISp tuft inputs. This stood in stark contrast to the ORB projection, for which the opposite was true, and apical regions of dense morphological overlap of axons and dendrites resulted in no functional input.

Next, we examined the correspondence between the anatomical input connectivity obtained from rabies tracing and the functional connectivity measured by total synaptic input. The number of rabies-labelled input neurons showed a strong contribution from RSPg and V2M, and modest input cell numbers for the more distal cortical regions (e.g., ACA, ORB). The total synaptic input, however, shows no correlation with these numbers (p = 0.8, r = -0.14, Spearman correlation, Figure 7B), with modest synaptic input from RSPg and most input arriving from V2M, ACA and ORB. Taken together, these results show clear specificity of dendritic targeting by brain-wide connections, with only a loose adherence to Peters’ rule for most inputs as well as large differences between anatomical and functional connectivity measured by rabies tracing and optogenetic stimulation, respectively.

## Discussion

Using data from an array of techniques for long-range circuit dissection, we carried out a direct assessment of Peters’ rule for brain-wide connections. Furthermore, we provide a comprehensive map of the dendritic targets of inputs to ttL5 neurons in the medial secondary visual cortex, V2M, in mice. Our recordings were targeted to V2M because cytoarchitectural definition of this region allows it to be identified in brain slices. One limitation of this approach is that V2M can be subdivided into separate areas using retinotopic maps (Garrett et al 2014, Zhuang et al 2017) or anatomical projection patterns (Gilissen et al 2021), which likely contribute to heterogeneity in connectivity. However, laminar distribution of axonal projections to VISpm, VISam and RSPagl from the input areas examined in this study are highly similar in the Allen Mouse Brain Connectivity Atlas (see Methods) and we observed no clear differences in sCRACM responses attributable to cell locations, supporting our working assumption of uniform longrange connectivity across V2M.

The whole-brain input map generated via rabies tracing was qualitatively similar to previous results from the primary visual cortex (Kim et al 2015). Axonal projections from the rabies-identified input regions broadly followed the expected pattern, with FB projections being biased toward L1 and FF toward the deeper layers (D’Souza et al 2020, Harris et al 2019, Rockland & Pandya 1979). Accordingly, L1 was densely innervated by higher-order areas like RSPg and ACA as well as the higher-order thalamic nuclei (LP, ATN). Interestingly, ORB, while forming part of the frontal association cortex, displayed a projection pattern attributed to FF areas and had projection neurons mainly in L2/3, another feature of FF connectivity.

Compared to the axonal projection patterns, synaptic input maps across input areas showed a remarkable degree of heterogeneity. One technical caveat of measuring distal inputs through somatic voltage clamp recordings is passive filtering along the dendrites. While this is difficult to correct for in an unbiased way, we minimized filtering by using a Cs^+^ based internal solution. Additionally, we quantified the integral, which is less affected by filtering, instead of the peak of the evoked currents. Importantly, our data showed no correlation between response magnitude and response location, arguing against large errors caused by dendritic filtering.

To test Peters’ rule, we compared anatomically predicted and functionally measured input maps. Several connections showed only weak correspondence between the two. For example, input from VISp, which is FF by definition and is assumed to primarily target perisomatic dendrites (Larkum 2013), was instead biased towards the apical tuft. Conversely, while the axonal projection pattern from ORB was almost identical, synapses formed almost exclusively with basal and oblique dendrites, indicating highly specific dendritic domain targeting. Other recorded areas (RSPg, ACA, ATN) partially conformed to Peters’ rule, yet still with significant differences between predicted and measured input distributions. The only area with strong adherence to Peter’s rule was LP. While it is possible that comparing individual axon, dendrite, and synaptic profiles on a single-cell basis would have given more accurate results, this was not possible as all three measures were not available for every neuron. However, the pattern of functional input from each input region was mostly consistent across cells. Morphologies of ttL5 neurons are likewise highly stereotypical and averaging is thus warranted. Furthermore, axon projection patterns used for evaluating Peters’ rule were measured from a subset of the same slices used for the sCRACM recordings, further supporting the direct comparison of predicted and functional input maps. The discrepancy resulting from averaging is thus likely to be low. Indeed, any smoothing resulting from averaging of morphologies, axonal projections or sCRACM maps across the areas comprising V2M would only increase overlap, and thus bias the results in favor of adhering to Peters’ rule.

Comparing anatomical connectivity obtained by rabies tracing to functional connectivity obtained by full-field optogenetic stimulation revealed large and unexpected differences. There are, however, technical caveats which might bias this comparison. First, while every rabies-identified area showed synaptic input when testing with sCRACM, the quantitative accuracy of rabies tracing is highly debated (Rogers & Beier 2021). Second, the magnitude of optogenetically evoked input depends on several technical parameters, such as infection efficiency and expression time. To facilitate comparison with rabies labelling, we maximized coverage for each input area by making several injections around the locations with highest rabies labelling density. Furthermore, there was no significant correlation between expression time and total input in any of the input areas (n = 7 areas, Benjamini-Hochberg correction with 0.1 FDR) and area identity explained more variance than expression time (r^2^ = 0.44 vs 0.11, n = 95 experiments). It is unlikely that these technical caveats alone could account for the remarkable discrepancy between anatomical and functional input magnitudes. There are several other possible explanations for this difference. First, axonal convergence may differ between input areas. For example, low convergence with one-to-one connectivity in inputs with strong rabies labelling could result in relatively weaker sCRACM input (VISp, RSPg). Meanwhile, strong synaptic currents relative to small rabies-labelled populations (ORB, ACA) may be explained by higher convergence. Such connections might be less discerning of their targets in order to convey more general contextual or state-specific information. Second, there may be a difference in the strength of individual synapses not reflected in rabies efficiency. A third contributing factor could be the recently reported activity-dependence of rabies transmission (Beier et al 2017). The apparent sparsity of some input areas (like ORB and ACA) could thus arise from having very low activity. Conversely, to result in extensive rabies labelling, VISp and RSPg should provide high-activity input.

It remains unclear what specific functions V2M serves. It has been linked to visual motion processing (Sun et al 2009) and navigation and spatial processing as part of the dorsal stream (Glickfeld & Olsen 2017, Powell et al 2020). However, little is known about what exact processing on what specific information is done in this area. This is in contrast to primary sensory cortical areas, such as VISp and SSp, where backpropagation activated calcium-spike (BAC) firing is a well-established dendritic mechanism linking apical tuft (assumed FF) and basal dendritic (assumed FB) inputs to support perception (Aru et al 2020). By contrast, ttL5 neurons in V2M do not show BAC firing (Galloni et al 2020), leaving local dendritic interactions to underlie their computational properties. While the exact computations performed are yet to be unraveled, a recent study showed that pyramidal neurons can learn complex nonlinear functions by exploiting local dendritic mechanisms to optimize synaptic weights (Bicknell & Hausser 2021). Knowledge of the dendritic distribution of input pathways thus provide key information for understanding which inputs are interacting locally during learning.

To explore the space of possible interactions between FF and FB inputs, we used a novel approach to allocate sCRACM input to specific dendritic compartments (Figure 7C). Thalamic FB input, which targets the apical tuft, arrives from multiple higher-order nuclei. Parts of ATN receive strong vestibular input (Rancz et al 2015) and together with RSP are critical for the head-direction system (Taube 2007, Velez-Fort et al 2018). Spatial and multisensory contextual information carried by LP inputs (Roth et al 2016) thus likely interacts with FF visual input in the tuft compartment. Unlike thalamic input, cortical FB inputs target all three dendritic domains. Surprisingly, the strongest of these, ORB and ACA, could interact with the local FF input at the level of oblique and basal dendrites. Frontal cortices are generally involved in decision making and motor control (Hamilton & Brigman 2015), and ORB additionally encodes spatial goals (Feierstein et al 2006). ACA, meanwhile, can regulate visual responses and sensory discrimination (Zhang et al 2014), and contributes to predictive learning in VISp (Fiser et al 2016). Their precise roles in the functioning of V2M, however, remains unknown. The strong input received by oblique dendrites is particularly important, as this compartment strongly affects L5 excitability (Schaefer et al 2003) and can gate information flow from the apical dendrites (Jarsky et al 2005). The particular targeting of ORB and ACA inputs to oblique dendrites might allow fine-level control of FF synapses at these dendrites, while simultaneously enabling them to exert gating control over tuft-projecting input from both the thalamic nuclei and VISp.

Overall, our results show that while FF or FB classification can be based on axonal projections (albeit with exceptions, such as ORB), macroscopic projectomes do not predict cell-type level input location. Consequently, there was no clear link between dendritic targeting and the organization of input areas in a cortical hierarchy. Furthermore, while rabies tracing is an effective tool to study general wiring diagrams, the proportion of input neurons thus estimated gives a poor estimate of functional input strength. Finally, the location and possible interactions between FF and the broad range of FB inputs, as well as their specific information content, suggests that ttL5 neurons may be adopting a multitude of integrative strategies that are more complex than previously believed.

## Figures and Tables

**Figure 1-1:** Number of rabies labelled cells in areas defined by the Allen CCFv3. Data deposited at https://github.com/ranczlab/Galloni.etal.2022

**Table 1:** Stereotaxic coordinates for viral injections. Distances in mm, AP from bregma, DV from pia. Square brackets denote a range [min, max].

area	AP	ML	DV	n. inj.
VISp	[-3.5, -2.8]	[1.8, 2.7]	[0.5, 0.6]	3
V2M	[-3.2, -2.7]	1	[0.2, 0.4]	2
RSPg	[-3.2, -2.7]	[0.4, 0.5]	[0.5, 0.7]	2
ACA	[0, 1.1]	[0.4, 0.5]	[1.2, 1.5]	3
ORB	[+2, +2.8]	1	[1.5, 2.3]	3
ATN	[-1.1, -0.5]	[0.5, 0.7]	[3.2,3.3]	2
LP	[-2.3, -1.7]	[1.4, 1.5]	[2.4, 2.6]	3

**Table 2:** Results of all sCRACM experiments.

input area	parameter	basal	oblique	tuft*		
**VISp**	peak location (μm)	-63	104	188	total input (pC)	0.93
N = 6	input proportion (%)	26%	33%	42%	total input SEM	0.11
n = 9	proportional input (pC)	0.24	0.30	0.39	soma depth (μm)	507 ± 22
	horizontal bias (μm) **	63	42	-21	cells with peak in tuft	5/9
**V2M**	peak location (μm)	-42	83	167	total input (pC)	11.24
N = 4	input proportion (%)	24%	62%	14%	total input SEM	1.56
n = 13	proportional input (pC)	2.68	6.96	1.60	soma depth (μm)	498 ± 15
	horizontal bias (μm) **	21	21	-21	cells with peak in tuft	1/13
**RSPg**	peak location (μm)	-42	42	125	total input (pC)	3.40
N = 9	input proportion (%)	30%	40%	30%	total input SEM	0.51
n = 20	proportional input (pC)	1.03	1.36	1.01	soma depth (μm)	503 ± 15
	horizontal bias (μm) **	21	21	0	cells with peak in tuft	2/20
**ACA**	peak location (μm)	-42	42	83	total input (pC)	9.46
N = 5	input proportion (%)	30%	45%	25%	total input SEM	1.32
n = 23	proportional input (pC)	2.83	4.22	2.41	soma depth (μm)	464 ± 9
	horizontal bias (μm) **	0	0	-21	cells with peak in tuft	1/23
**ORB**	peak location (μm)	-42	21	N/A	total input (pC)	7.16
N = 3	input proportion (%)	35%	57%	9%	total input SEM	1.43
n = 11	proportional input (pC)	2.47	4.05	0.64	soma depth (μm)	521 ± 19
	horizontal bias (μm) **	0	21	0	cells with peak in tuft	0/11
**ATN**	peak location (μm)	-104	104	104	total input (pC)	2.48
N = 3	input proportion (%)	8%	17%	75%	total input SEM	0.54
n = 8	proportional input (pC)	0.20	0.42	1.86	soma depth (μm)	435 ± 15
	horizontal bias (μm) **	21	42	21	cells with peak in tuft	6/8
**LP**	peak location (μm)	-42	21	63	total input (pC)	0.97
N = 4	input proportion (%)	10%	15%	75%	total input SEM	0.16
n = 10	proportional input (pC)	0.10	0.14	0.73	soma depth (μm)	500 ± 23
	horizontal bias (μm) **	-42	-21	-83	cells with peak in tuft	9/10
	**total proportional input**	**27%**	**49%**	**24%**	**sum total input (pC)**	**35.64**

* tuft measurements from pia, basal and oblique from soma

** negative distance means lateral, positive medial.

## Significance Statement

Perception and learning depend on the brain’s ability to shape neuronal representations across all processing stages. Long-range connections across different hierarchical levels enable diverse sources of contextual information, such as predictions or motivational state to modify feedforward signals. Assumptions regarding the organization of this hierarchical connectivity have not been extensively verified. Here we assess the synaptic connectivity of brain-wide projections onto pyramidal neurons in the visual cortex of mice. Using transsynaptic viral tracing and subcellular optogenetic circuit mapping, we show that functional synapses do not follow the consistent connectivity rule predicted by their axonal branching patterns. These findings highlight the diversity of computational strategies operating throughout cortical networks and may aid in building better artificial networks.
